# The impact of the COVID-19 pandemic on tuberculosis case reporting in Kazakhstan

**DOI:** 10.1093/eurpub/ckac130.194

**Published:** 2022-10-25

**Authors:** M Kulimbet, A Yergasen, N Glushkova, M Adenov, K Davletov

**Affiliations:** 1 Health Research Institute, Al-Farabi Kazakh National University, Almaty, Kazakhstan; 2 National Scientific Center of Phthisiopulmonology, Almaty, Kazakhstan; 3 Department of Epidemiology, Biostatistics and Evidence-Based Medicine, Al-Farabi Kazakh National University, Almaty, Kazakhstan

## Abstract

**Background:**

The COVID-19 pandemic had a significant impact on economic development, lifestyles and health systems in all countries. Due to the lack of medical interventions, many countries adopted restrictive measures to slow the spread of the virus and reduce the burden on the healthcare system. Quarantine measures have had an impact on the transmission of SARS-CoV-2. However, the unintended consequences of such drastic measures were inevitable. In developing countries, there have been adverse effects of disruptions in health services, including the provision of timely medical services in detecting cases of tuberculosis in the population. The aim of this study was to study the influence of COVID-19 pandemic on the tuberculosis incidence in the Republic of Kazakhstan.

**Methods:**

We analyzed national data on the reported tuberculosis cases and screening results of tuberculosis in Kazakhstan for 2019-2020. The primary data were collected from regular reporting of cases through surveillance.

**Results:**

The number of registered patients identified during screening activities in 2020 were 2,854 cases compared to 4,288 cases in 2019 before COVID-19 era. The proportion of cases with antibiotic-resistant (poly, multi, super) forms of tuberculosis increased up to 6.7% in 2020 in comparison with 2019.

**Conclusions:**

There is a need to conduct an analysis of the reasons for the increase in cases of multidrug-resistant and extensively drug-resistant tuberculosis. The working process should be adapted to epidemics and emergencies to ensure the availability of medical services, as well as to improve the system of preventive examinations and screening for the early detection of TB cases.

The abstract was submitted under the ‘CATINCA - Capacities and infrastructures for health policy development’ project which is coordinated/led by Robert Koch Institute and supported by the WHO Regional Office for Europe.

**Key messages:**

- The detection of tuberculosis during preventive examinations and screenings significantly decreased in 2020 compared to 2019.
- In recent years, there has been a significant increase in the number of cases with poly-, multi- and superantibiotic-resistant forms of tuberculosis.

